# Relationship between necrotic patterns in glioblastoma and patient survival: fractal dimension and lacunarity analyses using magnetic resonance imaging

**DOI:** 10.1038/s41598-017-08862-6

**Published:** 2017-08-16

**Authors:** Shuai Liu, Yinyan Wang, Kaibin Xu, Zheng Wang, Xing Fan, Chuanbao Zhang, Shaowu Li, Xiaoguang Qiu, Tao Jiang

**Affiliations:** 1 Department of Neurosurgery, Beijing Tiantan Hospital, Capital Medical University, Beijing, China; 2 Beijing Neurosurgical Institute, Capital Medical University, Beijing, China; 3 Brainnetome Center, Institute of Automation, Chinese Academy of Sciences, Beijing, China; 4 Department of Neuroradiology, Beijing Tiantan Hospital, Capital Medical University, Beijing, China; 5 Department of Radiation Oncology, Beijing Tiantan Hospital, Capital Medical University, Beijing, China; 6 Center of Brain Tumor, Beijing Institute for Brain Disorders, Beijing, China

## Abstract

Necrosis is a hallmark feature of glioblastoma (GBM). This study investigated the prognostic role of necrotic patterns in GBM using fractal dimension (FD) and lacunarity analyses of magnetic resonance imaging (MRI) data and evaluated the role of lacunarity in the biological processes leading to necrosis. We retrospectively reviewed clinical and MRI data of 95 patients with GBM. FD and lacunarity of the necrosis on MRI were calculated by fractal analysis and subjected to survival analysis. We also performed gene ontology analysis in 32 patients with available RNA-seq data. Univariate analysis revealed that FD < 1.56 and lacunarity > 0.46 significantly correlated with poor progression-free survival (*p* = 0.006 and *p* = 0.012, respectively) and overall survival (*p* = 0.008 and *p* = 0.005, respectively). Multivariate analysis revealed that both parameters were independent factors for unfavorable progression-free survival (*p* = 0.001 and *p* = 0.015, respectively) and overall survival (*p* = 0.002 and *p* = 0.007, respectively). Gene ontology analysis revealed that genes positively correlated with lacunarity were involved in the suppression of apoptosis and necrosis-associated biological processes. We demonstrate that the fractal parameters of necrosis in GBM can predict patient survival and are associated with the biological processes of tumor necrosis.

## Introduction

The World Health Organization (WHO) classifies astrocytomas into four grades on the basis of their histological features: WHO I, II, III, and IV^1^. Among these tumors, glioblastoma multiforme (GBM) is the most common and aggressive^2^. Compared with WHO I, II, and III gliomas, GBM exhibits necrosis as an important biological feature. Although the specific molecular events and pathways leading to its development remain unclear, the appearance of necrosis is associated with the tumor malignancy and patient prognosis. Therefore, the detection and evaluation of necrosis in GBM are important factors that need to be addressed in clinical practice.

In addition to histopathological examination, magnetic resonance imaging (MRI) is a convenient and noninvasive method for the detection of necrosis in GBM. In particular, post-contrast T1-weighted MRI can easily distinguish the necrotic area from the tumor mass. Hammoud *et al*. investigated the prognostic role of necrosis patterns in GBM using post-contrast T1-weighted MRI and reported that tumors with large areas of necrosis exhibited a poor prognosis^3^. Lacroix *et al*. evaluated 416 patients with GBM using the same method from the study by Hammoud *et al*. and reported significantly longer survival times in patients with small areas of necrosis who underwent aggressive resection^4^. Although these GBM studies placed emphasis on the importance of necrosis patterns on MRI, and their findings strengthen the overall understanding of necrosis in GBM, the precise evaluation of necrosis patterns remains difficult. Necrotic areas as observed on MRI generally exhibit irregular borders or high structural complexity, and this complicates segmentation and volume estimation. Till date, few studies have quantitatively analyzed the necrotic patterns of GBM on MRI. As a result, information masked by the structural complexity of the necrosis is often missed.

Fractal analysis is used to quantify natural objects with high structural complexities that are poorly represented by conventional Euclidean geometry^5^. Fractal dimension (FD) and lacunarity are two parameters used in fractal analysis to describe the complexity and distribution of a shape or subject. These parameters have also been used to distinguish different brain tumors^6^ and assign grades to gliomas^7^ in previous studies. In the present study, we used a semi-supervised learning algorithm to segment necrotic areas in GBM on post-contrast T1-weighted MRI and calculated FD and lacunarity values using fractal analysis. Then, we assessed the prognostic value of FD and lacunarity in GBM patients. Finally, to explore the molecular alternations and pathways leading to necrosis, we performed gene ontology (GO) analysis to investigated the role of lacunarity in the biological processes and pathways of necrosis in GBM. This is the first study quantitatively evaluating necrosis on MRI clinically and molecularly in GBM patients.

## Materials and Methods

### Patients

We retrospectively reviewed data for 95 patients with GBM that were collected from the Chinese glioma genome atlas (CGGA) database. The inclusion criteria were as follows: newly diagnosed lesions, no history of radiotherapy or chemotherapy, age > 18 years, a confirmed pathological diagnosis of supratentorial GBM, presence of necrotic regions in the tumors as observed on post-contrast T1-weighted MRI, and availability of follow-up data.

Clinical information was obtained from the CGGA database. The extent of surgical resection was determined by comparing pre- and postoperative magnetic resonance images. Gross total resection (GTR) was defined as the removal of all contrast-enhanced abnormalities. Partial resection was considered when GTR could not be achieved (<GTR). With regard to treatment, 86 of 95 patients (91%) had received radiotherapy and temozolomide, four (4%) had received temozolomide alone, and five (5%) had not received any adjuvant therapy. Progression-free survival (PFS) was calculated from the date of surgery to the date of tumor recurrence or the date on which the patient was last known to be progression-free. OS was calculated from the date of surgery to the date of death or the last follow-up. This study was approved by the Ethics Committee of Beijing Tiantan hospital, and carried out in accordance with the approved guidelines. All participants provided informed consent.

### MRI protocol

MRI was performed using a high-field 3.0 T MRI device (Siemens Trio, Siemens Healthcare, Germany). Both plain T1-weighted imaging [repetition time (TR), 450 ms; echo time (TE), 15 ms; section thickness, 5 mm] and post-contrast T1-weighted imaging (TR, 450 ms; TE, 15 ms; section thickness, 5 mm) with gadopentetate dimeglumine (Beilu Pharma, Beijing, China; 0.1 mmol/kg) were performed. The field of view was 24 cm and the matrix size was 256 × 256.

### Segmentation of the tumor and necrotic regions

All tumor lesions were manually delineated using MRIcro software (http://www.mccauslandcenter.sc.edu/mricro/). Tumor regions of interest (ROIs) were drawn by delineating the abnormal hyperintense signals on post-contrast T1-weighted images. Necrotic ROIs were defined as regions with decreased signal intensity within the tumor. Segmentation of necrotic ROIs was performed using a semi-supervised learning algorithm built in Matlab (R2014a, the MathWorks), which was developed by a neuroscientist in our team. The entire process of tumor and necrosis segmentation was conducted under the supervision of a senior neuroradiologist blinded to the clinical data of patients.

### Fractal analysis

**Figure 1:**
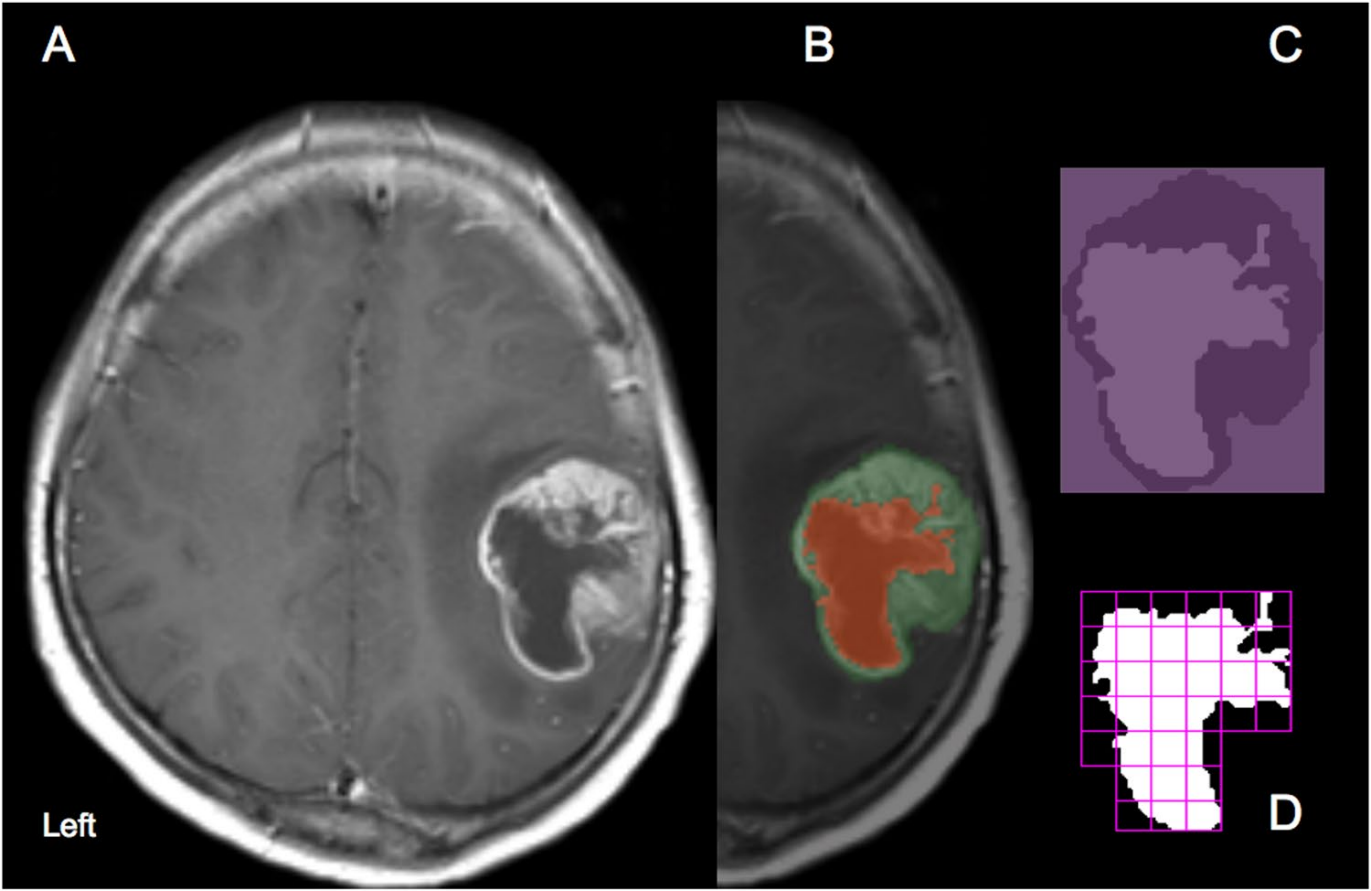
Segmentation and fractal analysis procedures for the analysis of necrotic patterns in glioblastoma. (**A**) A post-contrast T1-weighted magnetic resonance image of a representative patient with glioblastoma. (**B**) Segmentation of the tumor (green mask) and necrotic ROIs (red mask). (**C**) The necrotic ROIs are extracted with the tumor ROIs as the border. (**D**) Fractal analysis was performed by using the box-counting method.

Fractal analysis was performed using the Fraclac plug-in (Karperien–Charles Sturt University, Australia, https://imagej.nih.gov/ij/plugins/fraclac/FLHelp/Introduction.htm) of ImageJ (Rasband, W.S., ImageJ, U. S. National Institutes of Health, Bethesda, Maryland, USA, http://imagej.nih.gov/ij/, 1997–2016) software. Necrotic ROIs of the tumor were extracted with the tumor ROIs at the same slice as the border (Fig. 1). Following binarization, the two-dimensional data pertaining to the necrosis slices were loaded in ImageJ software. For fractal analysis, we used the box-counting method with Fraclac. The box sizes within the grids used ranged from a minimum of 2 pixels to a maximum of 45% of the image area. Twelve grid positions were used. FD and lacunarity were calculated for each grid and averaged to yield mean values. This method enabled us to determine FD and lacunarity values for all necrosis slices from tumors with varied numbers of slices. The mean FD and lacunarity values of all necrosis slices for each patient were used in further analysis.

### Survival analysis

Survival curves were generated using the Kaplan-Meier method and compared using log-rank tests. A Cox proportional hazards model was used to determine the independent prognostic values of FD and lacunarity. Considering the small number of factors under evaluation, Bonferroni correction was not further performed for the *p*-values.

### RNA sequencing data

RNA sequencing data for 32 patients was obtained from the CGGA database. Details regarding clinical specimen collection and the data-generating process have been described in our previous study^8^.

### GO analysis

To explore the role of lacunarity in the biological processes and pathways leading to necrosis in GBM, we screened the 32 patients with available RNA-seq data for genes correlated with lacunarity (Pearson R > 0.3). Genetic functions were analyzed using DAVID online tools (DAVID, https://david.ncifcrf.gov/).

### Statistical analysis

Gene data was processed by R language (https://www.r-project.org/) with a range of publicly available packages. A *p*-value of <0.05 was considered statistically significant.

## Results

### Demographic and clinical data

The clinical characteristics of the 95 enrolled patients are shown in Table 1. The median age was 50 years (range, 19 to 76 years), and 62% were men. The median preoperative Karnofsky performance score (KPS) was 70 (range, 50 to 100). GTR was peformed for 56% patients. The median follow-up period was 387 days, and 76 patients succumbed to the disease.

**Table 1:** Clinical characteristics of patients with GBM (n = 95).

Variables	Median (range)/Number (%)
Age	50 (19–76)
Gender	
Male	59 (62)
Female	36 (38)
Preoperative KPS score	70 (50–100)
Extent of surgery	
GTR	53 (56)
<GTR	42 (44)
Radiotherapy plus temozolomide	86 (91)
Fractal parameters	
FD	1.53 (1.29–1.69)
Lacunarity	0.46 (0.34–0.64)

Abbreviations: KPS = Karnofsky performance status; GTR = gross total resection; FD = fractal dimension.

### Fractal parameters

FD and lacunarity values are shown in Table 1. FD was negatively correlated with lacunarity (r = −0.658, *p* < 0.0001; Fig. S1).

### Survival analysis

**Figure 2:**
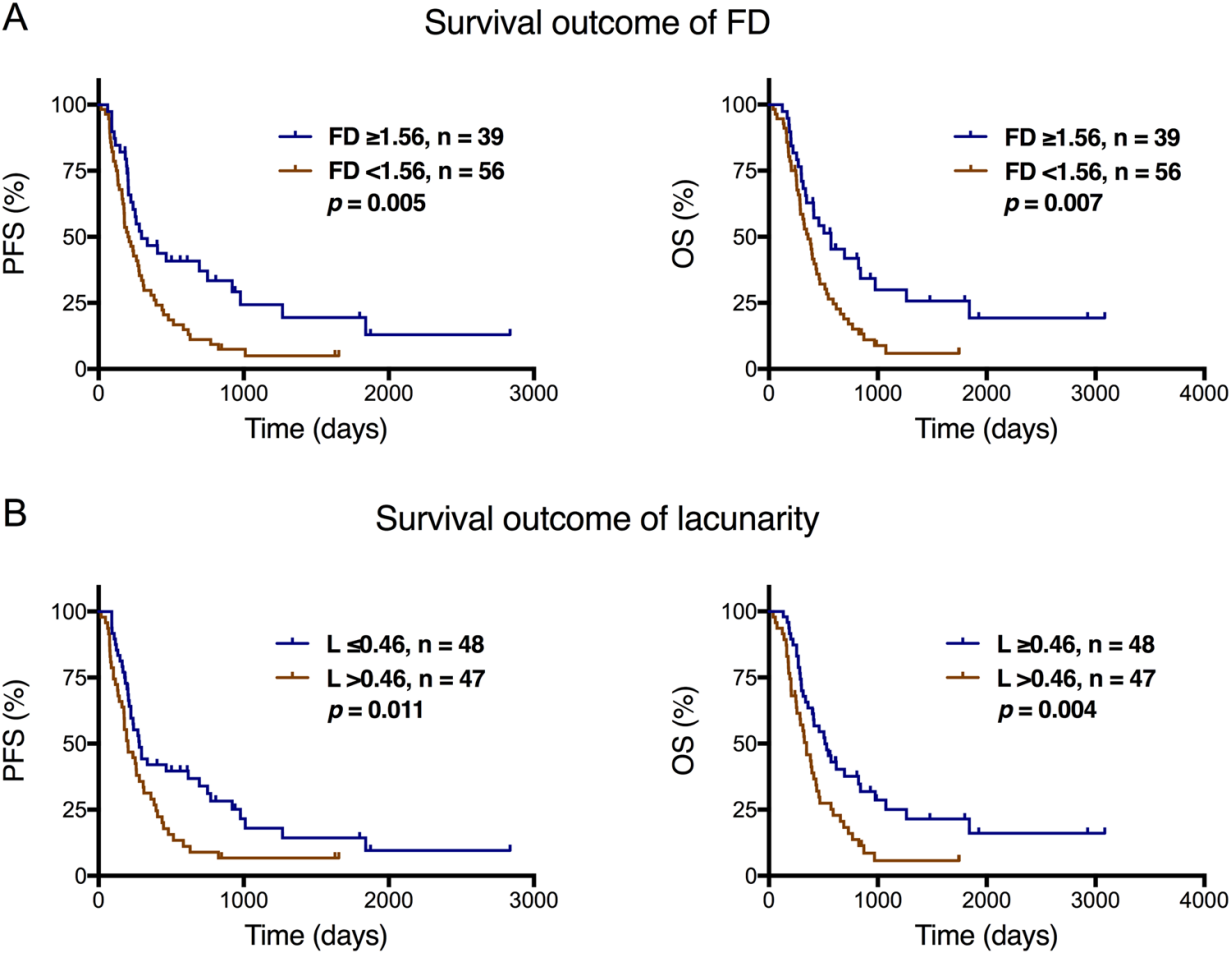
Kaplan-Meier curves showing the association of progression-free survival (PFS) and overall survival (OS) with the (**A**) fractal dimension (FD) and (**B**) lacunarity values.

In univariate Cox analysis, the clinical factors that were significantly associated with shorter PFS included age ≥ 50 years (*p* = 0.039), KPS < 80 (*p* = 0.015), partial resection (*p* = 0.031), FD < 1.56 (*p* = 0.006), and lacunarity > 0.46 (*p* = 0.012; Table 2). The clinical factors that were significantly associated with shorter OS included age ≥ 50 years (*p* = 0.014), KPS < 80 (*p* = 0.005), partial resection (*p* = 0.003), FD < 1.56 (*p* = 0.008), and lacunarity > 0.46 (*p* = 0.005; Table 2). To better demonstrate the prognostic role of FD and lacunarity, Kaplan-Meier curve estimates for PFS and OS are shown in Fig. 2.

**Table 2:** Univariate Cox analysis for factors potentially influence survival outcomes.

Characteristic	PFS			OS		
	p*	HR	95%CI	p*	HR	95%CI
Age ≥ 50	**0.039**	1.619	1.024–2.560	**0.014**	1.798	1.125–2.874
KPS < 80	**0.015**	1.766	1.115–2.796	**0.005**	1.998	1.233–3.237
Volume ≥ 50 (cm^3^)	0.250	1.299	0.832–2.027	0.227	1.326	0.839–2.094
< GTR	**0.031**	1.631	1.046–2.544	**0.003**	2.035	1.283–3.227
RT plus TMZ	0.266	0.672	0.333–1.354	0.267	0.658	0.315–1.377
FD < 1.56	**0.006**	1.933	1.209–3.091	**0.008**	1.919	1.182–3.117
Lacunarity > 0.46	**0.012**	1.774	1.133–2.777	**0.005**	1.935	1.220–3.068

Abbreviations: CI, confidence interval; HR, hazard ratio; RT, radiotherapy; TMZ, temozolomide; *without Bonferroni correction.

We further combined the fractal parameters with clinical factors including patients’ age, KPS, and resection extent that showed significant *p*-value in univariate survival analysis as prognostic signatures. Survival outcomes were compared based on these signatures, and the results showed better prognostic value than fractal parameters alone (Fig. S2).

Because FD and lacunarity were both fractal parameters that were significantly correlated with each other, we performed separate multivariate Cox analysis for the two factors. The results revealed that FD < 1.56 (*p* = 0.001) and lacunarity > 0.46 (*p* = 0.015) were both associated with shorter PFS and OS (*p* = 0.002 and *p* = 0.007, respectively; Table 3). Other clinical factors those were significantly associated with survival included age ≥ 50 years, partial resection, and KPS < 80 (Table 3).

**Table 3:** Multivariate Cox analysis for factors potentially influence survival outcomes (FD, lacunarity separately).

Characteristic	PFS			OS		
	*p*	HR	95%CI	*p*	HR	95%CI
**FD**						
Age ≥ 50	**0.032**	1.684	1.044–2.716	**0.012**	1.854	1.143–3.006
KPS < 80	0.189	1.374	0.855–2.210	0.175	1.417	0.856–2.346
Volume ≥ 50 (cm^3^)	0.465	1.202	0.734–1.966	0.584	1.152	0.694–1.911
<GTR	**0.043**	1.594	1.014–2.506	**0.005**	1.947	1.223–3.101
RT plus TMZ	0.656	0.846	0.406–1.764	0.376	0.705	0.325–1.528
FD < 1.56	**0.001**	2.171	1.348–3.496	**0.002**	2.145	1.314–3.502
**Lacunarity**						
Age ≥ 50	0.059	1.574	0.984–2.519	**0.020**	1.763	1.093–2.845
KPS < 80	**0.019**	1.737	1.095–2.756	0.069	1.592	0.965–2.626
Volume ≥ 50 (cm^3^)	0.618	1.133	0.694–1.851	0.852	1.049	0.631–1.744
<GTR	0.249	1.313	0.827–2.084	**0.017**	1.772	1.109–2.832
RT plus TMZ	0.398	0.733	0.357–1.505	0.261	0.645	0.300–1.386
Lacunarity > 0.46	**0.015**	1.746	1.114–2.736	**0.007**	1.891	1.186–3.014

### GO analysis

**Figure 3:**
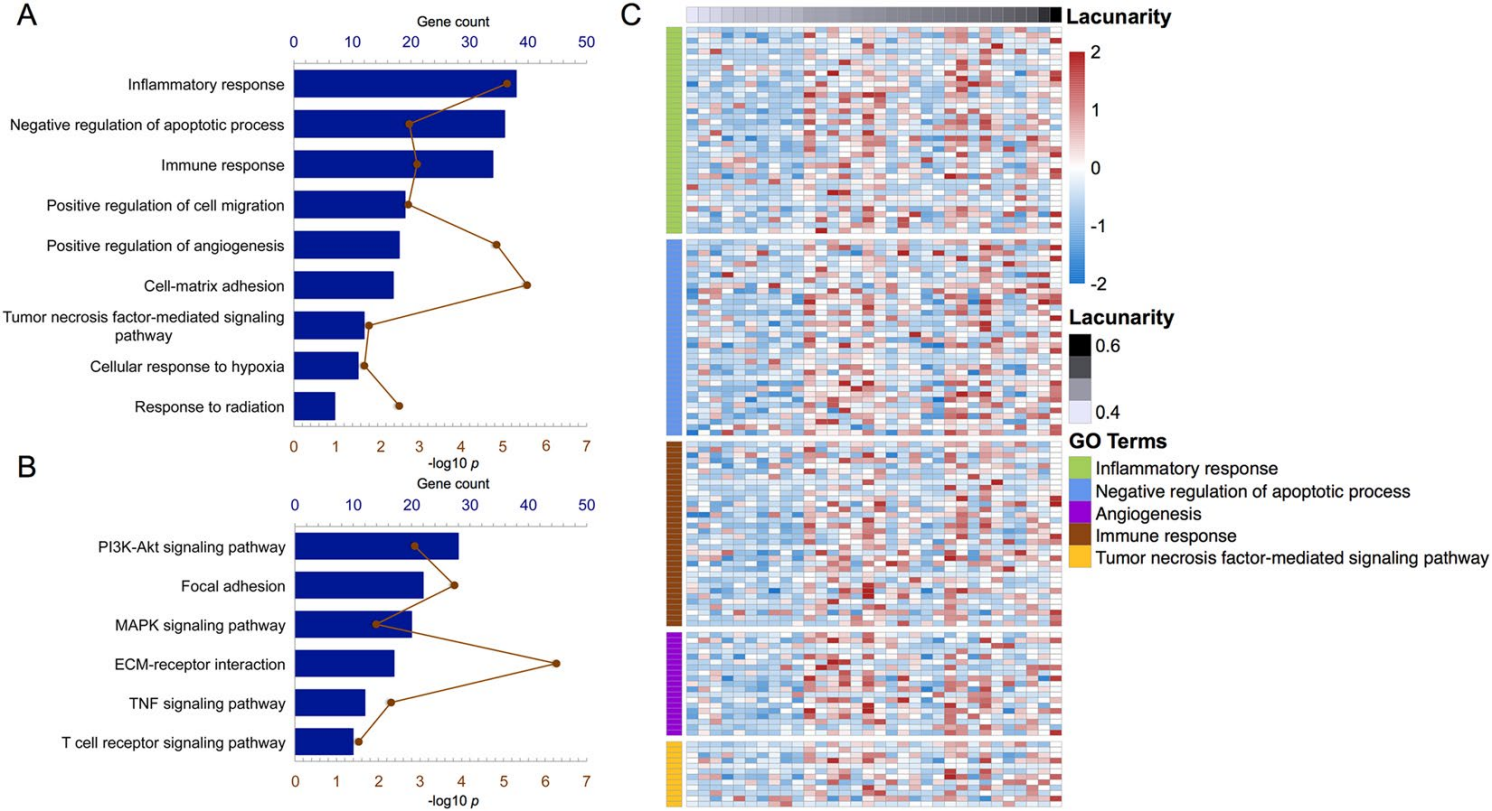
Gene ontology analysis for the lacunarity of necrosis in patients with glioblastoma. Biological processes (**A**) and pathways (**B**) associated with necrosis are shown. Genes involved in the representative biological processes are shown in (**C**).

GO analysis revealed that genes positively correlated with lacunarity were mainly enriched in three sets of biological processes: negative regulation of apoptosis and enhancement of inflammatory and immune responses; the tumor necrosis factor (TNF)-mediated signaling pathway; and malignancy-related processes, including cell-matrix adhesion, positive regulation of cell migration, positive regulation of angiogenesis, cellular response to hypoxia, and response to radiation (Fig. 3A). Moreover, these genes were mainly enriched in the PI3K-Akt signaling pathway, focal adhesion, mitogen-activated protein kinase (MAPK) signaling pathway, TNF signaling pathway, and T-cell receptor signaling pathway (Fig. 3B). The genes involved in the biological processes mentioned above are shown in Fig. 3C.

## Discussion

Although necrosis is a hallmark feature of GBM, it has not received adequate attention in clinical and scientific research. In the present study, we performed fractal analysis of MRI data pertaining to necrosis and determined the prognostic roles of FD and lacunarity. In addition, we used GO analysis explored the role of lacunarity in the biological processes and pathways of necrosis in GBM. Our findings emphasize the importance of necrosis in GBM with regard to patient prognosis and elucidate the alternations and molecular pathways leading to this necrosis.

Necrosis is generally complex in shape and shows large size variations, which complicate quantitative analysis. The first problem is delineation of the necrotic region in detail. With advances in soft computing, semi-automatic methods are becoming useful tools for brain tumor segmentation^9^. In the present study, we used a semi-supervised learning algorithm to segment necrosis in GBM. This algorithm is based on Gaussian fields and harmonic functions^10, 11^, and it aided in the accurate and efficient delineation of necrosis. The second problem is quantitative depiction of the characteristics of necrosis. Because of its irregular shape, volumetric analysis is nearly impossible. Fractal analysis employs a mathematical model that enables the measurement of irregular biological entities^12^, Application of the box-counting method^13^ facilitates the calculation of two fractal parameters, namely the FD and lacunarity. FD is an estimate of morphological complexity. More irregular objects exhibit higher FD values; thus, it provides a quantitative index of the roughness of natural objects^14^, Lacunarity is an index of measurement describing nonhomogeneity and translational and rotational invariance^15^. Images or patterns with higher lacunarity values exhibit higher heterogeneity and translational invariance^16^. Fractal analysis had been widely used in the field of neuroscience. In previous studies on brain tumors, FD and/or lacunarity values calculated from MRI data were used for tumor differentiation^6^ and glioma grading^7^. In the present study, we performed fractal analysis to quantitatively evaluate the complexity of necrosis.

The pattern and distribution characteristics of necrosis on MRI are associated with survival in GBM patients. In previous studies, a semi-quantitative method was used for evaluating necrosis in GBM on post-contrast T1-weighted MRI^3, 4^. According to the extent of necrosis, tumors were categorized into four grades. The results revealed that larger necrotic areas predicted a poor prognosis. In the present study, fractal analysis was performed for post-contrast T1-weighted MRI data. The results revealed that a lower FD value (<1.56) and a higher lacunarity value (>0.46) were significantly associated with poor PFS and OS. Thus, the prognostic role of necrosis patterns in GBM were demonstrated from a fractal standpoint. Considering that gaps always exist within or around the necrotic region, lacunarity may better reflect the heterogeneity and distribution characteristics of this entity. We also found that FD was negatively associated with lacunarity, a finding consistent with those of previous studies^17, 18^. Compared with FD, lacunarity alone may be enough to describe necrosis patterns and predict the survival of GBM patients.

Apoptosis and necrosis are the two major forms of cell death encountered in biology^19^. The process of necrosis has long been described as accidental and uncontrolled, although recent studies have shown that it is as well controlled and programmed as apoptosis. To investigate the biological processes and pathways of necrosis, we performed GO analysis for genes correlated with lacunarity. First, we found that genes positively correlated with lacunarity were more involved in the negative regulation of apoptotic processes, inflammatory responses, and immune responses. These functions reflect the particular biological behaviors of necrosis and indicate that it involves numerous inflammatory and immune responses^20^. whereas apoptotic cell death is inflammatorily and immunologically silent.

We further observed that lacunarity was positively correlated with the TNF-mediated signaling pathway. TNF plays a role in various biological processes, including immune and inflammatory responses. With regard to cancer, it plays a dual role in inducing cancer cell death in the form of apoptosis or necrosis^21^. GO analysis in the present study revealed that genes enriched in the TNF-mediated signaling pathway were also involved in the negative regulation of apoptosis and inflammatory and immune responses. In other words, genes positively correlated with lacunarity were associated with apoptosis suppression and necrosis promotion. This finding indicates that lacunarity can reflect the biology of necrosis.

Genes positively correlated with lacunarity were also enriched in the following processes: cell-matrix adhesion, positive regulation of cell migration, positive regulation of angiogenesis, cellular response to hypoxia, and response to radiation, all of which are associated with cancer cell invasiveness and resistance to treatment. These findings better our understanding of the prognostic role of necrosis lacunarity in GBM.

We also found that genes positively correlated with lacunarity were enriched in the PI3K-Akt and MAPK signaling pathways. Increased Akt activity can decrease the apoptotic potential of tumor cells, thus leading to necrosis^22^, while MAPKs are involved in the necrosis process and inflammatory response^23, 24^. We believe that these pathways may play important roles in necrosis development.

This study has some limitations. Using current MR technology, it is almost impossible to delineate the pathological boundary of a tumor lesion, if it exists. However, the structure of necrotic regions is much simpler than that of the tumor mass. The radiological characteristics of necrosis may be better representative of the pathological features. In addition, RNA-seq data were not available for all patients. Future prospective studies collecting more biological information are necessary.

In conclusion, our findings demonstrate the fractal features could reflect the biological characteristics of necrosis in GBM. The fractal parameters of FD and lacunarity can predict the survival of patients, while the parameter of lacunarity can reflect the biological processes and pathways leading to necrosis in GBM. These findings will aid in treatment planning for GBM patients and increase the existing knowledge base regarding necrosis in GBM.

## Electronic supplementary material

Supplementary material
