# Supplementary material for: Relationship between necrotic patterns in glioblastoma and patient survival: fractal dimension and lacunarity analyses using magnetic resonance imaging
Source: Sci Rep. 2017 Aug 16;7:8302. doi: 10.1038/s41598-017-08862-6 (PMC5559591; doi:10.1038/s41598-017-08862-6)
Supplement: Supplementary file 1 — Supplementary material [file 41598_2017_8862_MOESM1_ESM.doc]

**Supplementary material**

**Relationship between necrotic patterns in glioblastoma and patient survival: fractal dimension and lacunarity analyses using magnetic resonance imaging**

Shuai Liu, Yinyan Wang, Kaibin Xu, Zheng Wang, Xing Fan, Chuanbao Zhang, Shaowu Li, Xiaoguang Qiu and Tao Jiang

**
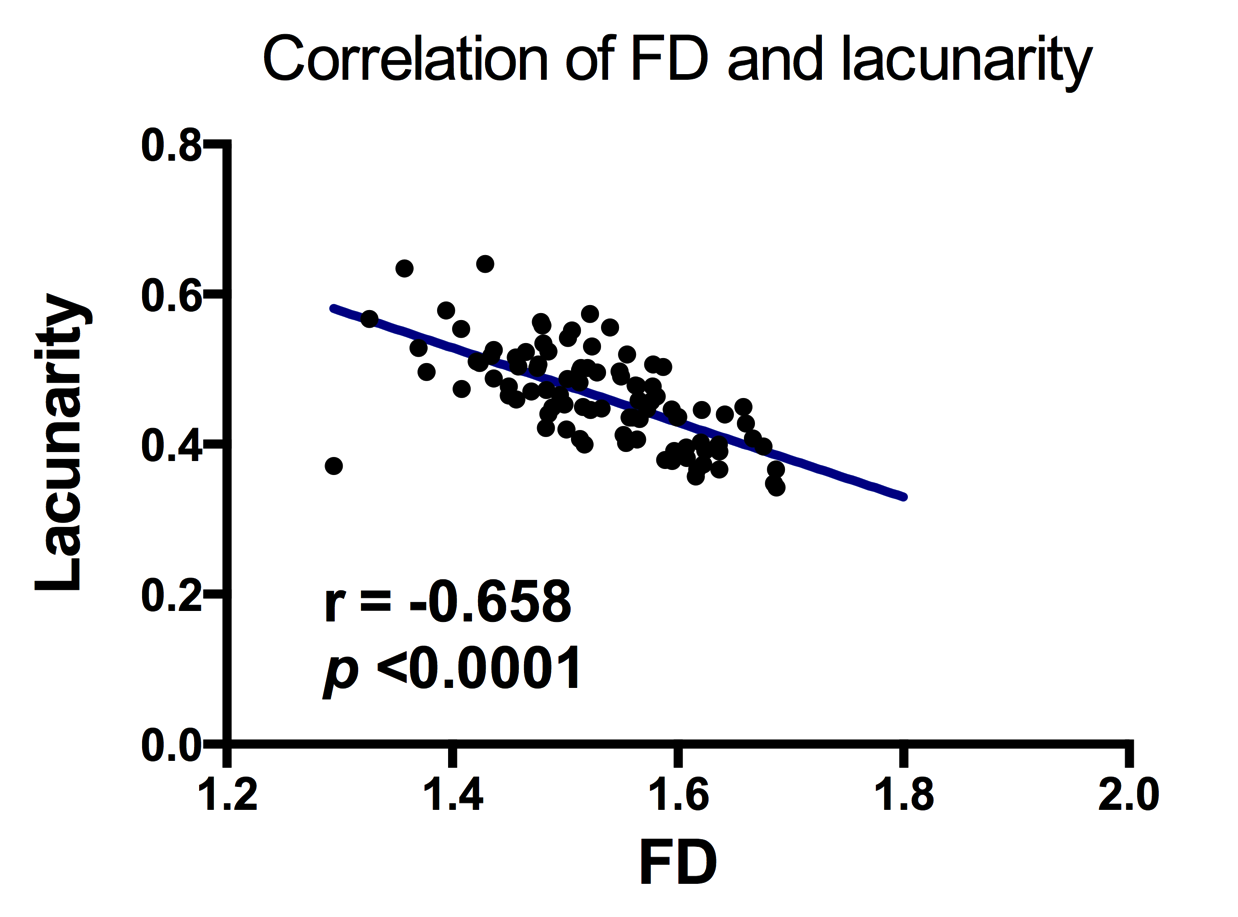
**

**Figure S1. Correlation between the fractal dimension (FD) and lacunarity.**

A significant negative correlation is observed.


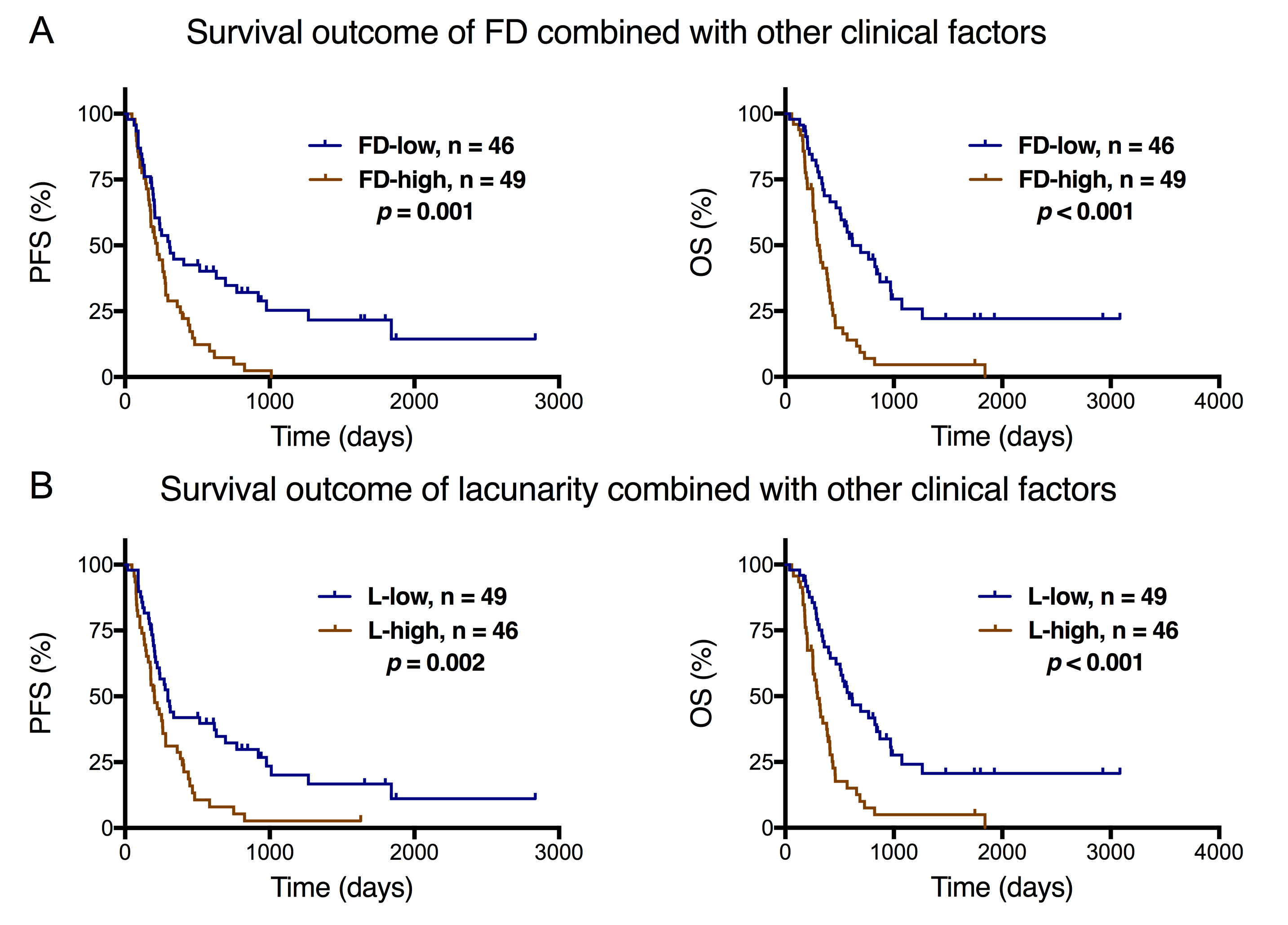


**Figure S2. Kaplan-Meier curves based on survival signatures.**

(A) Survival signature of fractal dimension (FD) combined with other clinical factors. FD-low and FD-high indicate groups separated by low and high scores. (B) Survival signature of lacunarity (L) combined with other clinical factors. L-low and L-high indicate groups separated by low and high scores.
